# Supplementary material for: The Prophylactic Effects of Glutamine on Muscle Protein Synthesis and Degradation in Rats with Ethanol-Induced Liver Damage
Source: Nutrients. 2021 Aug 14;13(8):2788. doi: 10.3390/nu13082788 (PMC8398394; doi:10.3390/nu13082788)
Supplement: Supplementary file 1 [file nutrients-13-02788-s001.zip › nutrients-1322168-supplementary.pdf]

Supplement 1 Effects of glutamine supplementation on plasma and hepatic triglyceride levels in chronic ethanol-fed rats[1,2]

Group	Plasma (mg/dL)	Liver (mg/g liver)
CC	54.0 ± 24.6	12.0 ± 1.9
CE	48.1 ± 16.4	15.3 ± 0.5
GG	36.4 ± 13.8	14.6 ± 0.7
GE	46.6 ± 30.1	14.0 ± 1.5
GEG	31.5 ± 16.2	13.0 ± 1.0

[1]Values were expressed as the mean ± SD.

[2] The CC group was fed the control diet for 8 weeks; the GG group was fed a glutamine-containing diet for 8 weeks; the CE group was fed the control diet the first 2 weeks and then an ethanol-containing diet for the next 6 weeks; the GE group was fed a glutamine-containing diet the first 2 weeks and then an ethanol-containing control diet for the next 6 weeks; the GEG group was fed a glutamine-containing diet for the first 2 weeks, and then a glutamine-containing diet with ethanol for the next 6 weeks.

Supplement 2 Effects of glutamine supplementation on amino acid composition in rats chronically fed ethanol[1,2]

	Blood (μM)					Liver (μM)					Muscle (μM)				
	CC	CE	GG	GE	GEG	CC	CE	GG	GE	GEG	CC	CE	GG	GE	GEG
**Val**	166.37 ± 18.43	165.09 ± 32.31	137.09 ± 13.83	370.88 ± 232.93	310.77 ± 231.09	683.10 ± 4.49^{a}	463.85 ± 57.48^{b}	643.03 ± 23.60^{a}	662.39 ± 12.56^{a}	623.46 ± 80.80^{a}	144.04 ± 18.56	117.75 ± 18.65	130.98 ± 120.27	111.25 ± 16.78	89.22 ± 24.06
**Tyr**	145.71 ± 13.85	154.80 ± 13.36	154.11 ± 68.90	150.67 ± 42.24	147.55 ± 6.10	400.49 ± 37.62	484.23 ± 26.76	395.50 ± 100.87	432.19 ± 69.50	502.28 ± 33.43	163.56 ± 23.11	174.87 ± 82.70	151.15 ± 128.32	182.98 ± 43.29	213.23 ± 76.24
**Thr**	0.35 ± 0.60	0.06 ± 0.11	0.17 ± 0.29	0.00 ± 0.00	0.21 ± 0.37	9.48 ± 3.22^{b}	33.98 ± 23.68^{a}	4.68 ± 2.40b	4.82 ± 2.91b	7.36 ± 2.96b	0.00 ± 0.00	0.39 ± 0.57	1.26 ± 1.90	1.04 ± 1.35	0.29 ± 0.51
**Ser**	0.00 ± 0.00	0.00 ± 0.00	0.00 ± 0.00	0.00 ± 0.00	0.00 ± 0.00	0.00 ± 0.00	0.00 ± 0.00	0.00 ± 0.00	0.00 ± 0.00	0.00 ± 0.00	0.00 ± 0.00	0.00 ± 0.00	11.87 ± 20.55	0.04 ± 0.06	0.00 ± 0.00
**Pro**	24.04 ± 1.63^{b}	25.92 ± 4.91^{ab}	23.91 ± 0.47^{b}	24.67 ± 0.77^{b}	29.84 ± 1.47^{a}	158.11 ± 19.35^{b}	227.65 ± 48.30^{ab}	317.12 ± 6.66^{a}	297.63 ± 49.09^{a}	223.29 ± 91.17^{ab}	21.26 ± 1.37^{b}	19.26 ± 1.00^{b}	34.42 ± 13.23^{a}	17.28 ± 2.36^{b}	15.77 ± 2.99^{b}
**Phe**	159.75 ± 23.48^{ab}	153.97 ± 18.72^{ab}	136.89 ± 4.29^{b}	154.48 ± 16.65^{ab}	176.98 ± 5.32^{a}	598.32 ± 0.58^{ab}	592.16 ± 2.62^{bc}	596.40 ± 2.99^{abc}	590.87 ± 5.82^{c}	598.82 ± 3.60^{a}	279.25 ± 26.64	186.05 ± 60.65	255.66 ± 230.49	213.48 ± 73.23	137.81 ± 22.67
**Lys**	1.57 ± 0.39	3.34 ± 2.86	1.79 ± 0.60	2.69 ± 0.72	2.66 ± 0.90	12.90 ± 3.69^{c}	18.95 ± 5.50^{ab}	22.81 ± 0.53^{ab}	22.54 ± 3.39^{ab}	29.78 ± 14.38^{a}	6.39 ± 1.49	4.01 ± 0.27	7.97 ± 4.46	5.50 ± 1.92	6.19 ± 2.06
**Leu**	165.78 ± 53.98	294.45 ± 113.12	298.90 ± 72.42	169.77 ± 37.03	230.88 ± 74.04	558.39 ± 11.11	543.53 ± 201.17	622.77 ± 77.04	674.87 ± 58.40	614.07 ± 111.96	322.07 ± 17.74^{a}	254.93 ± 151.22^{b}	206.54 ± 143.45^{b}	522.34 ± 117.05^{a}	385.48 ± 215.84^{a}
**Ile**	227.68 ± 41.22	249.81 ± 77.59	293.00 ± 22.15	215.63 ± 33.52	286.91 ± 87.46	668.15 ± 45.00^{b}	613.80 ± 13.65^{c}	725.33 ± 3.15^{a}	701.76 ± 8.82^{ab}	731.92 ± 43.65^{a}	235.60 ± 22.26	237.08 ± 79.68	225.30 ± 171.59	257.01 ± 82.55	227.51 ± 55.77
**Gly**	0.00 ± 0.00	0.00 ± 0.00	0.00 ± 0.00	0.00 ± 0.00	0.00 ± 0.00	0.00 ± 0.00	0.00 ± 0.00	0.00 ± 0.00	0.00 ± 0.00	0.00 ± 0.00	0.00 ± 0.00	0.00 ± 0.00	5.79 ± 10.03	0.00 ± 0.00	0.00 ± 0.00
**Gln**	7.22 ± 0.58^{a}	6.41 ± 0.57^{a}	6.05 ± 0.94^{a}	3.74 ± 1.44^{b}	5.61 ± 0.85^{b}	22.29 ± 12.53^{a}	11.18 ± 2.52^{b}	16.63 ± 7.21^{b}	10.53 ± 5.38^{b}	32.36 ± 10.06^{a}	10.96 ± 4.08	5.55 ± 2.00	23.70 ± 27.99	5.39 ± 0.50	8.32 ± 1.73
**Glu**	1.44 ± 0.36	1.37 ± 0.32	1.02 ± 0.22	1.02 ± 0.25	1.13 ± 0.19	60.70 ± 5.08^{a}	38.50 ± 7.71^{b}	56.11 ± 10.58^{a}	47.84 ± 6.75^{ab}	52.23 ± 11.73^{ab}	7.43 ± 2.92^{ab}	5.24 ± 1.18^{ab}	10.77 ± 7.05^{a}	3.77 ± 0.99^{b}	4.33 ± 0.81^{b}
**Asp**	0.00 ± 0.00	0.03 ± 0.06	0.00 ± 0.00	0.00 ± 0.00	0.00 ± 0.00	61.85 ± 8.72^{ab}	51.78 ± 20.04^{a}	68.96 ± 4.36^{ab}	72.95 ± 7.13^{a}	72.58 ± 5.59^{a}	1.60 ± 0.56^{ab}	1.53 ± 0.06^{ab}	4.73 ± 4.71^{a}	0.21 ± 0.08^{b}	1.41 ± 1.59^{ab}
**Asn**	0.00 ± 0.00	0.00 ± 0.00	0.00 ± 0.00	0.00 ± 0.00	0.00 ± 0.00	0.00 ± 0.00	0.00 ± 0.00	0.00 ± 0.00	0.00 ± 0.00	0.00 ± 0.00	0.00 ± 0.00	0.00 ± 0.01	0.00 ± 0.00	0.00 ± 0.00	0.00 ± 0.00
**Arg**	2.67 ± 0.57^{a}	1.24 ± 1.11^{b}	2.25 ± 0.31^{ab}	1.48 ± 0.71^{ab}	2.32 ± 0.35^{ab}	0.25 ± 0.17	0.39 ± 0.04	0.27 ± 0.25	0.19 ± 0.04	0.17 ± 0.05	2.00 ± 0.44	1.97 ± 0.61	5.14 ± 5.81	1.22 ± 0.11	1.28 ± 0.62
**Cys**	0.00 ± 0.00	0.00 ± 0.00	0.00 ± 0.00	0.00 ± 0.00	0.00 ± 0.00	0.00 ± 0.00	0.00 ± 0.00	0.00 ± 0.00	0.00 ± 0.00	0.00 ± 0.00	0.00 ± 0.00	0.00 ± 0.00	0.00 ± 0.00	0.00 ± 0.00	0.00 ± 0.00
**His**	0.00 ± 0.00	0.00 ± 0.00	0.00 ± 0.00	0.00 ± 0.00	0.00 ± 0.00	28.00 ± 9.00	28.81 ± 2.83	36.26 ± 0.76	27.96 ± 17.23	29.57 ± 21.59	7.36 ± 5.05	5.67 ± 4.12	8.35 ± 1.27	7.83 ± 0.86	9.64 ± 1.20
**Ala**	0.00 ± 0.00	0.00 ± 0.00	0.00 ± 0.00	0.00 ± 0.00	0.00 ± 0.00	3.66 ± 0.28	4.75 ± 2.69	3.79 ± 0.16	4.39 ± 1.17	4.51 ± 1.24	1.52 ± 0.16	1.87 ± 1.07	1.31 ± 0.28	1.21 ± 0.18	2.60 ± 2.04
**Met**	14.34 ± 12.12	9.95 ± 7.20	7.52 ± 4.11	7.86 ± 0.74	13.98 ± 2.74	200.79 ± 87.97	82.78 ± 38.89	181.73 ± 50.97	148.29 ± 32.43	183.06 ± 101.58	27.58 ± 0.57	29.72 ± 1.72	28.85 ± 22.65	24.16 ± 3.17	23.43 ± 5.09
**Try**	20.93 ± 4.17	17.62 ± 5.27	15.22 ± 1.69	17.87 ± 3.86	17.82 ± 0.04	20.47 ± 1.13^{a}	12.24 ± 1.20^{c}	17.33 ± 1.57^{ab}	16.36 ± 1.06^{b}	19.35 ± 3.09^{ab}	10.50 ± 0.42^{ab}	13.82 ± 7.18^{ab}	8.20 ± 4.66^{b}	9.34 ± 0.13^{ab}	19.12 ± 10.09^{a}

[1]Values are expressed as the mean ± standard deviation. Means with different superscript letters in the same row significantly differ ($p<0.05$).

[2] The CC group was fed the control diet for 8 weeks; the GG group was fed a glutamine-containing diet for 8 weeks; the CE group was fed the control diet the first 2 weeks and an ethanol-containing diet the next 6 weeks; the GE group was fed a glutamine-containing diet the first 2 weeks and then an ethanol-containing diet the next 6 weeks; the GEG group was fed a glutamine-containing diet the first 2 weeks, and then a glutamine-containing diet with ethanol the next 6 weeks. Val, valine; Tyr, tyrosine; Thr, threonine; S, serine; Pro, proline; Phe, phenylalanine; Lys, lysine; Leu, leucine; Ile, isoleucine; Gly, glycine; Gln, glutamine; Glu, glutamic acid; Asp, aspartic acid; Asn, asparagine; Arg, arginine; Cys, cysteine; His, histidine; Ala, alanine; Met, methionine; Try, tryptophan.
